# Three-Dimensional Culture Systems for Dissecting Notch Signalling in Health and Disease

**DOI:** 10.3390/ijms222212473

**Published:** 2021-11-19

**Authors:** Guya Diletta Marconi, Cristina Porcheri, Oriana Trubiani, Thimios A. Mitsiadis

**Affiliations:** 1 Orofacial Development and Regeneration, Institute of Oral Biology, University of Zurich, 8032 Zurich, Switzerland; GUYA.MARCONI@unich.it (G.D.M.); cristina.porcheri@zzm.uzh.ch (C.P.); 2 Department of Medical, Oral and Biotechnological Sciences, University “G. d’Annunzio” Chieti-Pescara, 66100 Chieti, Italy; oriana.trubiani@unich.it

**Keywords:** spheroids, organoids, organ-on-a-chip, microfluidics, 3D culture systems, Notch signalling, cancer, drug screening, regeneration, therapy

## Abstract

Three-dimensional (3D) culture systems opened up new horizons in studying the biology of tissues and organs, modelling various diseases, and screening drugs. Producing accurate in vitro models increases the possibilities for studying molecular control of cell–cell and cell–microenvironment interactions in detail. The Notch signalling is linked to cell fate determination, tissue definition, and maintenance in both physiological and pathological conditions. Hence, 3D cultures provide new accessible platforms for studying activation and modulation of the Notch pathway. In this review, we provide an overview of the recent advances in different 3D culture systems, including spheroids, organoids, and “organ-on-a-chip” models, and their use in analysing the crucial role of Notch signalling in the maintenance of tissue homeostasis, pathology, and regeneration.

## 1. Introduction

In vitro cultures have been established over a century ago and are largely used for studying physiological and pathological conditions, modulation of signalling pathways, and gene expression modifications. Cells derived from normal and/or diseased tissues can be grown either as adherent monolayers or in suspension. These two-dimensional (2D) tissue cultures only partially recapitulate the cellular behaviour of the tissue of origin, as they cannot reproduce complex cell–cell and cell–extracellular matrix interactions. This prompted the development of three-dimensional (3D) cell culture systems, where the existing interactions between the various cell populations in the tissue of origin are largely maintained [1,2,3,4]. Recently, 3D culture systems have been further improved, by incorporating structural tissue elements, including components of the extracellular matrix (ECM). Cells can be grown in 3D culture settings that support the formation of tissue-specific spheroids and organoids, as well as in more complex organotypic models (e.g., “organ-on-a-chip”). These models provide a unique platform to dissect the molecular communication between cells and, ultimately, the role of molecular pathways in the regulation of tissue homeostasis.

## 2. The Notch Signalling Pathway

**Figure 1:**
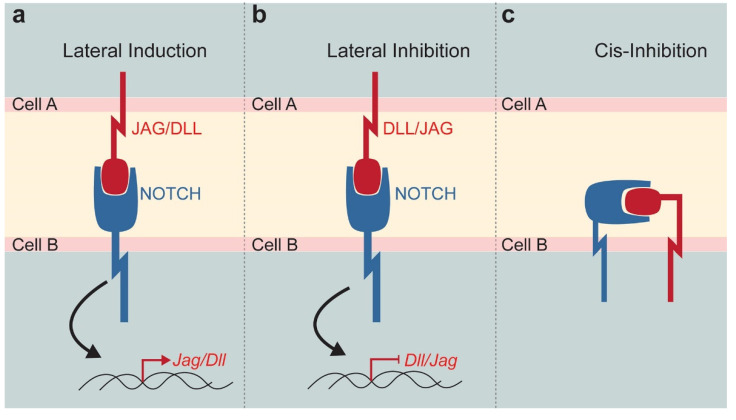
Schematic representation of the Notch signalling function. Notch pathway modulation strongly depends on the Notch receptor—DLL/JAG ligand interaction established between neighbouring cells. (**a**) Lateral induction triggers the expression of the *Jag* or *Dll* in the receiving cell. (**b**) Lateral inhibition reduces the expression of *Dll* or *Jag* in the neighbouring cell. (**c**) Cis-inhibition occurs when receptor and ligand expressed by the same cell, activates the pathway and sequesters active molecules on a single cell surface. Abbreviations: DLL, Delta-like; JAG, Jagged.

Notch signalling is one of the major pathways controlling cell fate specification, intercellular communication, tissue organisation, and morphology [5,6,7,8]. The canonical Notch signalling is a cell–cell communication mechanism, where juxtaposed cells physically interact via transmembrane receptors and ligands exposed on opposite cell membranes [9,10,11]. Four type of Notch receptors (Notch1, Notch2, Notch3, and Notch4) and five Notch ligands (Jagged1 and Jagged2, Delta-like1, Delta-like3, and Delta-like4) have been described in mammals. These molecules share a high degree of sequence homology with their *Drosophila* counterpart, which is an indicator of a highly conserved pathway throughout evolution. The interaction between receptors and ligands can give rise to either a lateral induction or a lateral inhibition, with respective activation or inhibition of ligand expression in the neighbouring cells [12]. Notch–ligand interaction triggers a cascade of protein cleavages, leading to the release of the active Notch intracellular domain (NICD). NICD translocates to the nucleus where it forms a transcriptional complex with Suppressor of Hairless (or RBP-Jk or CSL), Mastermind and transcriptional co-activators to modulate the expression of Notch-downstream target genes (mainly Hes and Hey families), ultimately resulting in the regulation of the Notch ligand transcription (Figure 1a,b). This signalling cascade is relatively simple since only few proteins are involved in the Notch pathway. However, the combinations of interactions between the four different ligands and the five different receptors might generate downstream diversity with distinctive signalling outputs. Although this is not yet well-studied, several findings suggest differences among ligands and receptors, as well as diversity of post-translational modifications of Notch receptors [13]. Non-canonical, CSL-independent NICD activity and ligand-independent activation of Notch signalling have been also reported [13]. In the case of lateral induction, the expression of the ligand in the receiving cell increases, while a lateral inhibition reduces the expression of the same ligand [10,14]. Finally, receptors and ligands can be expressed on the same cell, reducing their availability for interactions with the neighbouring cell. This cis-inhibition is a fine tuning of the Notch signalling that results in a general inhibition of the pathway (Figure 1c) [15].

During organogenesis, the Notch pathway is involved in cell fate determination and the formation of tissue boundaries [16]. During adulthood, Notch signalling plays an important role in regulating the behaviour of stem cells, by maintaining their stemness and influencing their fate in most tissues [17,18]. Notch signalling is active in various adult stem cell niches [19]. For example, interaction between Notch1 and Delta-like4 (Dll4) is necessary for establishing hematopoietic clusters in the aorta-gonad-mesonephros, which is the primordial niche for hematopoietic stem cells [20]. Similarly, Notch signalling is primordial for neural stem cells maintenance during both the embryonic and adult life [21,22,23]. Neural stem cells expressing Notch keep their undifferentiated status, while absence of Notch induces their differentiation [23,24]. Indeed, adult stem cells of the dentate gyrus are maintained in a semi-quiescent state via the Notch1-Hes5 axis, and depletion of *Notch1* results in a reduced number of neurons [25].

A number of diseases are linked to mutations in the Notch signalling pathway [13,26]. NOTCH3 mutations in humans cause the Cerebral Autosomal Dominant Anteriopathy with Subcortical Infracts and Leukoencephalopathy (CADASIL), a disease associated with recurring small brain infarcts and degeneration of vascular smooth muscle cells in the brain. Mutations in the JAGGED1 (JAG1) ligand and more rarely in the NOTCH2 receptor cause the Alagille syndrome that affects multiple organs, including heart, liver, kidney, and craniofacial organs. Furthermore, NOTCH1 mutations are linked to aortic valve diseases, and DELTA-LIKE3 (DLL3) mutations to spondylocostal dysostosis [13]. The Notch pathway is also involved in the generation of cancers [27]. Frequently, components of the Notch signalling are not mutated in most cancers. However, NOTCH1 mutations have been detected in patients with acute lymphoblastic leukaemia (T-ALL) tumours, as well as in patients with both, small cell lung cancer (SCLC) and non-small cell lung cancer (NSCLC). Furthermore, NOTCH1 mutations in the skin lead to the generation of skin cancer, while mutations in the hematopoietic compartment lead to myeloproliferative disorders [26,28]. In addition, upregulation of NOTCH1 and/or JAG1 can lead to various pathologies, such as breast cancer [29] and prostate cancer [30]. These findings suggest that Notch signalling disbalance plays a very important critical role in the generation and progression of a disease. 

Since Notch signalling plays a major role in organogenesis, pathology, and regeneration, different 3D culture platforms such as spheroids, organoids, and “organ-on-a-chip” devices can be used to dissect the roles and the regulation of this pathway in various healthy and pathological cell populations. The fine tuning of the levels of Notch signalling may provide new therapeutic scenarios for the various Notch-linked diseases, and this possibility could be fully investigated and explored in the above-mentioned 3D culture systems.

## 3. 3D Culture Systems

3D culture systems provide the new means of generating tissues in a more physiological manner. However, these systems never fully recapitulate heterogeneity and the complexity of the organs and tissues, since they lack the fluctuations that exist in vivo. Furthermore, the major components of 3D systems, such as cell input, ECM parameters, protein concentrations, are combined in a manner that represents the simplified version of the in vivo unit. Here, we present the most used 3D culture systems, namely the spheroids, organoids, and “organ-on-a-chip” devices. 

### 3.1. Spheroids

**Figure 2:**
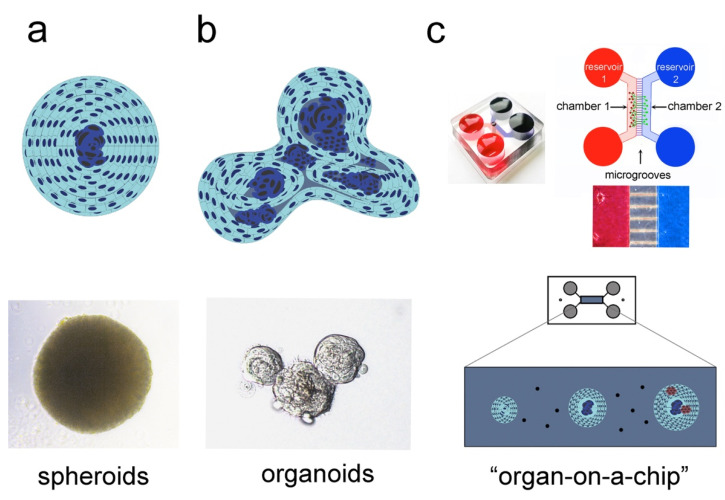
3D culture systems used to recapitulate the in vivo tissue organisation and complexity. Simplified overview of three different culture models: spheroids (**a**), organoids (**b**), and “organ-on-a-chip” (**c**). (**a**) Spheroids represent the simplest 3D culture system. Spheroids constitute efficient model systems for studying cell–cell communication, cellular metabolism, oxygen gradients, and nutrients distribution. Their complexity can be scaled up by integrating into the spheroids different cell types, such as stem cells and endothelial cells, thus providing the ideal platform for biochemical and molecular testing, as well as rapid drug screening. (**b**) Organoids reproduce the complexity of the native tissue, where cells at various cytodifferentiation states, ranging from stem cells and progenitors to fully differentiated cells, coexist. (**c**) “Organ-on-a-chip” closely mimics several aspects of the native organ. Integrated into a microfluidic system, 2D cultures, spheroids and organoids can be exposed to a dynamic microenvironment that allows controlled fluid exchange and interactions between different tissues, such as vessels and nerves (adapted from [64,65]).

Isolated cells cultured in low-attachment wells naturally aggregate to form 3D microtissues, known as multi-cellular spheroids. This mechanism of cell self-assembly has been initially observed in sponges, where upon their dissociation, cells were able to aggregate and recreate a new sponge body [31,32]. More recent studies have shown that pluripotent stem cells (PSCs) derived from murine blastocyst were also able to aggregate, thus forming the embryoid bodies (EBs), which can be maintained as 3D structures in vitro [33]. This simplified 3D culture system allowed the investigation of complex cellular and molecular mechanisms occurring during embryo growth. Intercellular communication and differentiation inputs were preserved in the EBs, thus modelling the development of the three germ cell layers and their reciprocal interaction (i.e., ectoderm, mesoderm, and endoderm) [34,35]. Similarly, Chinese hamster V79 lung cells grown as single cell suspension were able to aggregate and form spheroids that are characterised by a hierarchical structure, where proliferating cells occupy the most peripheral parts and quiescent cells the central part (core) of the spheroids [36]. This spheroidal organisation broadly mirrors the structural complexity of living tissues, where different cell types position themselves according to their needs for oxygen, nutritional gradients, and interactions with other subgroups of cells. Therefore, multicellular spheroids preserve the important biological properties of the tissue of origin, providing higher cell viability, stable morphology, and physiological metabolic activity (Figure 2a) [2,37,38]. 

The 3D structure of spheroids provides the ideal setting for studying the fine regulation of Notch signalling and the precise Notch ligand–receptor interactions. Endothelial cells have been successfully co-cultured with specific mesenchymal stem cell (MSCs) populations for the formation of mixed spheroids. These spheroids demonstrate an increased survival rate that correlates with upregulation of Notch expression [39]. More particularly, the vasculogenic potential in mixed spheroids appears to be Notch3-dependent, as Notch3 deletion blocks the sprouting of new forming vessels [40]. Although nutrients and oxygen are easily distributed in the external layers of the spheroids, these elements are less accessible in their core. Cells situated in the core of the spheroids react to these hypoxic conditions by activating the Notch pathway, which in turn correlates with the upregulation of hypoxia-inducible factor-1 (HIF-1), hence triggering angiogenesis [40,41].

### 3.2. Organoids

Organoids are generated from adult stem cells (ASCs) and/or PSCs of various tissues and organs. In these 3D cultures, the differentiation program of the tissue of origin is maintained, and a simplified miniature version of the organ develops in vitro (Figure 2b). Organoids can acquire a spherical shape in the initial phase, but their further growth leads to the establishment of more complex 3D structures that closely resemble the organ of origin [37,42,43]. Organoids have been successfully generated from brain, intestine, heart, thymus, liver, lung, and pancreas [31,37,43,44,45,46,47,48]. Therefore, organoids are well-accepted model systems for studying stem cell behaviour in pathophysiological conditions, and further analyse cell–cell communication and the cells–ECM interactions. Unlike spheroids, which develop in the absence of scaffold, the development and survival of organoids often requires the presence of various biomimetic materials (e.g., Hydrogel, Matrigel) [49,50]. Organoids are more complex than spheroids, but largely maintain basic features of the tissue of origin by recapitulating its essential function and spatially-restricted cell lineage commitment [50,51].

Modulation of Notch signalling in organoids might influence the fate and behaviour of cells, as well as the crosstalk between stem cells and stroma cells, thus recapitulating the in vivo situation. Indeed, the oscillatory expression patterns of the Notch effector Hes1, reported in embryonic stem cells (ESCs) and during neurogenesis, is maintained in cortical organoids generated from induced PSCs (iPSCs) [52]. Cell differentiation is disrupted when Hes1 oscillations cease, which suggests that tissue homeostasis and stem cell maintenance are strictly controlled by the Notch signalling [53,54,55].

### 3.3. “Organ-on-a-chip” Technology

Spheroids and organoids represent an important step forward in the reproduction of the in vivo physiology, although major elements of complexity are still lacking (e.g., lack of innervation, absence of fluid perfusion to create a dynamic environment, inefficient nutrient and waste transport, etc.) [42,43,44,55,56,57]. Organoids can be placed in microfluidic chambers that work as an irrigation system allowing continuous flow of nutrients, thus mimicking the vascular system by carrying nutrients and oxygen [56,58]. Spheroids/organoids grown in a microfluidic chamber face physical constraints, including shearing forces, compression-induced stretches and stiffness variations in the ECM [59,60,61]. These biomechanical cues reflect the in vivo stimuli and contribute to create more accurate copies of the native organs [61,62,63]. Drugs, chemical products, and signalling molecules can be evenly distributed in the chamber where cells are grown, thus emulating the biological tissue reactions upon the various pharmaceutical treatments in vivo (Figure 2c) [64]. Different cell types from a given tissue/organ can be co-cultured in these microfluidic devices, thus establishing the inter-cellular crosstalk that guarantees tissue functionality. “Organ-on-a-chip” devices are already available for various organs and tissues, including kidney, bone, cartilage, skin, and ovary [65].

Innervation in microfluidics can be simulated upon co-culture of ganglia or single neurons with spheroids/organoids, where the establishment of neuronal connections with specific cell populations can be studied in detail [66]. The interactions between neurons and cells might define the fate of cells and affect their cytoskeleton restructuring [55]. Microelectrode arrays have been also used in microfluidic constructions for the simulation of the electrical activity of the neuronal network [63,67].

The infusion of components of the immune system in the microfluidic systems could be useful for studying tissue reactions under pathological conditions [68,69,70]. The Notch pathway is central in the regulation of the immune system [71,72]. Notch signalling, which is directly involved in the maturation of lymphoid organs, is activated during pro-inflammatory response by cytokines such as Tumour Necrosis Factor (TNF) and Interleukin-1 (IL1), and regulates the myelomonocytic differentiation via inhibition of Hes1 [73,74,75,76,77].

## 4. Modelling the 3D Microenvironment

In all living organisms, cells dynamically interact with their environment, thus receiving trophic support and directional instructions that are important for tissue/organ maintenance. Cells aggregated in spheroids or grown as organoids preserve these interactions and therefore the crosstalk between the various cell populations, as well as between the cells and their specific microenvironments can be easily studied [60,78,79]. However, the 3D systems are designed in a simplified manner compared to human tissues and organs, in order to have an efficient and easy way to answer specific questions. Therefore, many of the parameters, which normally exist in the microenvironment of the human organs, are not reproduced in the 3D systems. Positioning of cells within the 3D structures follows a hierarchical distribution that can recreate the precise and complex pattern of a given tissue [80,81]. Advanced imaging systems combined with modern immunodetection techniques explore the spatial distribution of cells expressing exclusive molecular markers that allows defining the cellular organisation of a 3D structure. This information is important since the special distribution of cells and their structural polarity is paramount for the functionality of specialised tissues and organs [60,82,83]. Recently, the combination of 3D cultures with next-generation sequencing (NGS) techniques, such as single cell RNA sequencing, and spatial transcriptomics, advanced our knowledge on tissue composition, greatly assisting in the identification of fundamental molecules during tissue development, pathology, and regeneration [79,83,84,85]. The chemical and physical structure of the ECM used in 3D cultures confers the unique environment to instruct the renewal, proliferation, and differentiation of cells. This is well established, since depletion of such microenvironmental cues (e.g., fibronectin, collagen, and laminin) during embryonic or post-natal life severely compromises the morphology and function of tissues and organs [61,86,87,88,89,90]. Therefore, artificially fabricated scaffolds or substrates that mimic ECM composition of specific tissues offer appropriate homing to the cells. This opens a multitude of possibilities for analysing various parameters that influence cell behaviour and functionality, such as the stiffness, biodegradability and porosity of scaffolds, and the application of physical forces [78,91]. Cell–cell communications and cell–ECM interactions play a fundamental role in the stability and integrity of all tissues [87]. Modelling their structures and their cellular composition based on the detection of specific molecular markers could improve our understanding in tissue pathology and progression of a disease. For example, Notch signalling has a significant role in the epithelium and changes in the Notch activity often result in dysplastic tissue formation [92,93]. During embryonic development, epithelial structures express both Notch ligands and Notch receptors [94]. Notch signalling is involved in the establishment of the epithelial stem cell niches through regulation of *Tp63* and *Keratin14* expression in keratinocyte progenitor cells [80,95,96]. Alterations in the Notch pathway have been identified in carcinomas, such as colorectal cancers, breast cancers, squamous cell carcinomas, and lung adenocarcinomas [92,93,97]. Therefore, 3D models could be powerful tools to study the effect of pharmacological modifications of Notch signalling and their consequences in tissue homeostasis.

## 5. 3D Systems in Regenerative Medicine

The recent developments in 3D culture systems open new horizons in medical fields relying on the therapeutic potential of stem cells. Stem cells require specific microenvironmental cues to preserve their self-renewal capacities and undifferentiation status. 3D cultures have been established with a variety of stem cell types, using ESCs, ASCs and iPSCs, among others [43,44]. Stem cells grown in organoids preserve their self-renewal characteristics, the expression of molecules linked to stemness, and their ability to progress into various specific cell fates [90,98]. Adult bone marrow was the initially identified source of MSCs [99]. Over the last decades, MSCs populations were isolated from many other tissues and organs, such as the adipose tissue, periosteum, trabecular bone, synovium, skeletal muscle, and teeth [83,100,101]. MSCs isolated from human teeth can be grown in 3D spheroids, and are able to differentiate into many cell types, such as osteocytes, chondrocytes, adipocytes, and neuronal cells [21,83,100,101,102,103]. Both in vitro and in vivo studies have demonstrated that the osteogenic potential of dental stem cells (DSCs) cultured as spheroids has improved, when compared to that of the 2D cultured DSCs [17,104,105,106]. Similarly, in vitro studies have shown a higher cell differentiation potential in spheroids than in 2D culture systems [107]. To analyse the in vivo regenerative/healing potential of spheroids and organoids, these 3D structures can be implanted in pathological and defective tissues of various animal models. Upon implantation into the malfunctioning tissue, stem cells from the 3D structure could initiate the healing process and contribute to the complete tissue restoration, as this was the case for the intestinal tissue [39,48,108]. 3D structures generated by patient-derived stem cells, can also be used for analysing and testing various pharmaceutical products (i.e., drug screening), thus establishing the basis for future regenerative personalised treatments [51].

These novel tissue engineering techniques, combined with modern genetic and pharmacological tools provide unprecedented powerful solutions in the field of regenerative medicine for the repair of damaged tissues. However, important limitations still exist for transferring these techniques in the clinics. Implantation of any sort of undifferentiated material into the live tissues, for therapeutic purposes, is not yet free of side-effects and carries an enormous risk of uncontrolled cell proliferation and cancer initiation [109,110,111]. Therefore, a deep knowledge on the signalling molecules involved in the establishment of the stem cells niches in the 3D structures is necessary in order to overcome these limitations. The maintenance of the stem cell niches is complex and requires the interaction between molecules of different signalling pathways [18,112]. Accessible in vitro systems mimicking the characteristics and structure of stem cell compartments of various organs have been already used for studying the role of Notch signalling in stem cell populations [15]. The interaction between Wnt and Notch signalling has been described in human iPSC-derived cortical spheroids, where inhibition of either one or both pathways can affect brain tissue identity [113].

## 6. 3D Cultures in Disease Modelling

Pathological conditions arise from structural and functional alterations of the organ. Cells from pathological tissues can be used in 3D systems for analysing the molecular and cellular mechanisms involved in these pathological processes. Additionally, bioengineered 3D models can be used for testing drug toxicity and efficacy, a first step in the development of novel pharmacological products. Albeit partially, several pathophysiological conditions such as wound healing processes, inflammatory diseases and cancers are currently reproduced in 3D cultures [114,115,116,117,118,119]. We here present three systems as a proof of principle for the usage of 3D culture in disease modelling. 

***Skin wound healing*** is a complex process involving vascularisation, stem cell migration, immunoreaction, growth factors release, and synthesis of ECM [120]. All these processes have to be strictly synchronised. Spheroids containing specific cell populations can be transplanted into wounded tissues, thus greatly improving the healing process, due to the release of growth factors, induced immunomodulation, and targeted integration to the damaged tissue [121,122]. In vitro skin models have been developed from keratinocytes and fibroblasts co-cultures, grown in specific ECM scaffolds [114]. Air–liquid interface adds to the complexity of these 3D structures that represent accurate models for skin wound healing therapies [114]. Notch signalling is activated during wound healing, while its blockage results in important delay of tissue regeneration. On the other hand, induction of its activity enhances tissue repair [123]. Similarly, modulation of Notch affects keratinocyte behaviour in scratch assays in vitro [123,124]. 3D cultures can therefore be used to analyse the roles of Notch molecules during the various stages of skin regeneration and wound healing [123].

***Chronic liver diseases***, where the inflammatory processes derive from infections or continuous exposure of chemicals, are often reproduced in vitro using patient-derived cells or cell lines [125,126]. However, primary cells are viable for only a limited number of passages, while immortalised cell lines may not completely mirror the physiological conditions [127]. Liver-derived organoids bypass these limitations, allowing for the maintenance of hepatoblasts in culture for longer time [128]. In contrast to classical hepatocyte monolayer models, the 3D culture system does not result in cell dedifferentiation and changes in their metabolic activities [127,129,130]. Hence, 3D spheroids from hepatic cells constitute interesting tools for studying chronic and acute conditions in liver diseases [131]. Genetic defects of Notch pathway components result in severe liver malformations. For example, mutations of the *Jagged1* gene result in bile ducts reduction and consequent dysfunction of the biliary tree [132,133]. Hence, the generation of organoids from healthy and pathological liver tissues provides an additional tool for further exploring the potential therapeutic roles of Notch signalling [134].

***Cancer*** is one of the most common disease models that can be reproduced in 3D cultures. Cancer establishes a self-protective environment that allows aberrant cells to access nutrients and resources, to ultimately expand and self-renew while escaping the immune surveillance of the host [135]. This specialised microenvironment can be partially reproduced in cancer-derived spheroids [136]. Most cancers have a component of undifferentiated cells, known as cancer stem cells, which maintain the tumour core and allow seeding of metastasis in distant locations [136,137,138]. Organoids and spheroids derived from cancer tissues represent excellent platforms for drug screening and personalised medicine. These 3D systems allow for expansion of the initially limited pool of cancer cells, thus recapitulating essential tumour features [139]. Cancer-derived spheroids or organoids can be also studied in microfluidic “organ-on-a-chip” devices, allowing a more accurate and complete investigation [3,140,141]. The Notch pathway can play both an oncogenic and anti-tumorigenic role, depending on the type of cancer and the tissue involved. Notch signalling regulation during neo-angiogenesis plays a crucial role in restructuring the tumorigenic microenvironment, influencing oxygen and nutrients income [142]. Additionally, the role of Notch in immunomodulation has a great impact in the tumorigenic growth [72]. The generation of tumours strongly depends on specific cell–cell communications and cell–ECM interactions. 3D culture models are of great help in order to understand tissue dysfunction and cancer formation due to aberrations in Notch signalling [138,143,144]. Neuroblastoma and breast cancer-derived spheroids showed upregulation of Notch expression, concomitant to the increase of cells that are positive to cancer stem cell markers [145,146]. Notch1 and Notch2 deletions lead to structural alterations in cutaneous squamous cell carcinoma [147,148]. Intestinal tumoroids are also used to study the effects of aberrant Notch signalling in the generation of colorectal cancers [149,150,151,152,153].

## 7. Drug Screening and Therapy Testing in 3D Culture Systems

**Figure 3:**
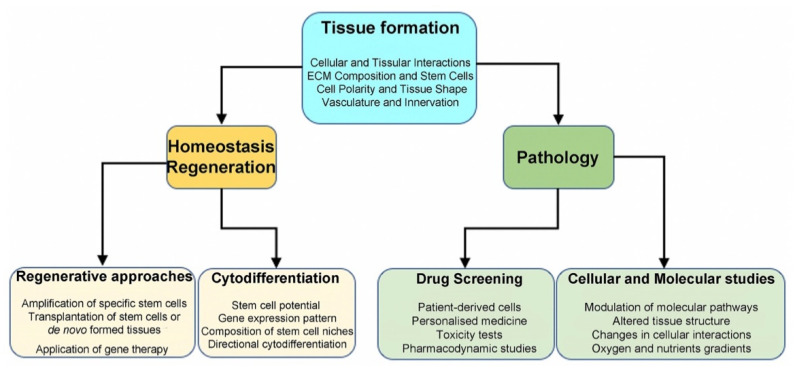
Summary of 3D systems and their applications. Several aspects of tissue organisation can be modelled in 3D cultures. For all applications, spheroids, organoids, and “organ-on-a-chip” represent more faithfully the main features of native tissues when compared to 2D cultures. 3D systems allow characterisation of specific stem cell populations and could be used for regenerative purposes. Furthermore, 3D structures can reproduce a disease environment and serve for drug testing.

Drug discovery is a multifaceted, time-consuming, risky, and expensive process, which aims at the identification of novel molecules with therapeutic potential. Newly developed molecules might present unexpected and unwanted effects during the clinical trial phases, that could be associated with the limitations of current preclinical models [154]. The conventional 2D cell culture systems do not accurately simulate the in vivo tissue reactions in both physiological and pathological conditions, thus limiting their value to study pharmacokinetics and pharmacodynamics of new drugs [4]. Instead, 3D culture models have gained attention in recent years in the pharmacological field, including the drug discovery field, mainly due to their capability to emulate faithfully the in vivo testing conditions [154]. The advantages of 3D models to simulate the characteristics of the native tissue, promoted their applicability in clinical testing, drug screening, disease modelling, and in predicting the acquisition of drug resistance [1,155,156]. Spheroids and/or organoids allow accurate evaluation of cell alterations due to oxygen, growth factors, nutrients, and drug modifications [157]. Spheroids and organoids can be used in personalised medicine for high-throughput screening of pharmacological responses. Microfluidic devices assess more faithfully drug concentrations, but several caveats still remain in translating the results to in vivo settings [156,158,159]. It is therefore obvious that new important information is collected in recent years by combining tissue, genetic and molecular engineering technologies and “organ-on-a-chip” platforms [160,161]. For example, main features of the “organ-on-a-chip” technology, such as the vascular support, the tissue–tissue communication and the use of different cell types, contributed to more detailed and valid studies on drug effects (in tissues or single cells) [161,162]. These 3D microfluidic devices are also used to predict the pharmacological reactions in humans, ultimately reducing the usage of animal models and clinical trials [162,163,164] (Figure 3).

Notch pathway deregulation creates severe pathological conditions, including cancer and cardiovascular diseases, and therefore constitutes a promising target for novel pharmacological treatments [144]. Blockage of the Notch activity can be achieved by interference between receptor–ligand interaction (e.g., via monoclonal antibodies), or by preventing receptor cleavages (GSI-DAPT molecules) [144,165]. The efficiency of these approaches is strongly limited by severe side effects, mainly due to systemic off-target effects [165,166,167]. Thus, developing 3D in vitro models that preserve the tissue complexity might constitute a safe way for molecular analysis and understanding the big potential of Notch signalling modulation in disease treatment.

## 8. Conclusions

3D culture systems that faithfully mimic the in vivo environment and cell behaviour are excellent simulation models for physiological and pathological conditions. These technologically advanced tools are extremely useful for studying specific molecular cues and analysing the role of specific signalling pathways, including the Notch pathway, in the context of specific disease models, drug responses and tissue regeneration.

## Data Availability

Not applicable.

## Abbreviations

NICD	Notch Intracellular Domain
Dll4	Delta-like4
3D	Three-dimensional
EBs	Embryoid Bodies
PSCs	Pluripotent Stem Cells
MSCs	Mesenchymal Stem Cells
HIF1	Hypoxia inducible factor 1
ASCs	Adult Stem Cells
ECM	Extracellular Matrix
ESCs	Embryonic Stem Cells
iPSC	Induced-Pluripotent Stem Cells
TNF	Tumour Necrosis Factor
IL1	Interleukin-1
NGS	Next Generation Sequencing
DSCs	Dental Stem Cells
